# Loneliness and Neurocognitive Aging

**DOI:** 10.20900/agmr20210009

**Published:** 2021-03-29

**Authors:** R. Nathan Spreng, Danilo Bzdok

**Affiliations:** 1 Laboratory of Brain and Cognition, Montreal Neurological Institute, Department of Neurology and Neurosurgery, Faculty of Medicine, McGill University, Montreal, QC H3A 2B4, Canada; 2 Departments of Psychiatry and Psychology, McGill University, Montreal, QC H3A 2B4, Canada; 3 Douglas Mental Health University Institute, Verdun, QC H4H 1R3, Canada; 4 McConnell Brain Imaging Centre, Montreal Neurological Institute (MNI), McGill University, Montreal, QC H3A 2B4, Canada; 5 Department of Biomedical Engineering, Faculty of Medicine, McGill University, Montreal, QC H3A 2B4, Canada; 6 School of Computer Science, McGill University, QC H3A 2A7, Canada; 7 Mila-Quebec Artificial Intelligence Institute, Montreal, QC H2S 3H1, Canada

**Keywords:** loneliness, social isolation, aging, dementia, Alzheimer’s disease, social cognition, MRI, default mode network

## Abstract

Loneliness imposes significant risks to physical, mental and brain health in older adulthood. With the social distancing regimes implemented during the COVID-19 pandemic, there is even greater urgency to understand the human health costs of social isolation. In this viewpoint we describe how the experience of loneliness may alter the structure and function of the human brain, and how these discoveries may guide public health policy to reduce the burden of loneliness in later life.

In times of human crisis, personal and communal resilience depends on strength of our social connections [1]. Perceived social isolation, or loneliness, arises when desired social needs go unmet. Loneliness has been considered an aversive yet adaptive evolutionary signal, akin to hunger, that promots greater social integration [2]. Even before the worldwide COVID-19 emergency, public health agencies were warning that combating loneliness is among the greatest health challenges of our time [3]. Loneliness is estimated to affect 10–20% of adults in many Western countries who say that they often or always feel lonely, lack companionship, feel left out, or isolated from others [4]. This prevelance soars in older adults, with 30–40% of older individuals reporting the experience of loneliness [5,6].

The health consequences of loneliness are pervasive, affecting not only the mind, but the entire human body. The experience of loneliness is associated with physical health morbidities including hypertension and immune system dysfunction [7], as well as higher mortality through suicidality [4]. A sense of loneliness has been associated with greater health risks than obesity, or smoking 15 cigarettes daily [8]. Self-reported loneliness has also been related to susceptibility to major psychiatric disorders [4], cognitive decline [9] and increased dementia risk in later life [10-12]. Controlling for demographic, somatic, and psychiatric risk factors, including anxiety and depression, lonely individuals have been found to be 1.64 times more likely to develop clinical dementia than persons who do not self-report as lonely [13]. Consistent with this finding, there is now substantial evidence that loneliness is associated with an acceleration in Alzheimer’s disease (AD) neuropathology [14-16]. In short, loneliness in older adulthood imposes clear risks to physical, mental and brain health.

Human and non-human animal studies have now begun to elucidate the impact of loneliness on brain structure and function [1]. Yet uncertainty surrounds the precise biological mechanisms which link loneliness in humans to normative brain aging and neurodegenerative disease. Recent work from our laboratories is beginning to shed light on this question. We have identified the default network as a core substrate of perceived social isolation and loneliness in the human brain [17,18]. The default network is a collection of functionally connected regions located principally along the brain’s midline as well as regions of the medial temporal lobes including the hippocampus [19]. These regions show close spatial overlap with the “social brain” [20,21] and are known to be strongly implicated in social abilities [22,23]. Importantly, the default network is selectively vulnerable to age-related decline and AD pathology [24]. We argue that the default network provides a candidate neural substrate linking loneliness, aging, and brain health across the lifespan.

To directly interrogate this hypothesis, we have begun to harness large cohort data resources to identify associations between the experience of loneliness and brain structure and function. In an earlier study, we reported, in a sample of younger adults, that increased loneliness was associated with a shift in the functional connectivity patterns of the default network, towards greater functional integration with networks implicated in externally directed perceptual, attentional and cognitive control functions [17]. We speculated that these findings speak to the evolutionary hypothesis [2], suggesting that loneliness is an adaptive signal, prompting a shift away from internal mentation towards a more external focus promoting social re-engagement.

We have recently built on these early hints with the largest-to-date study of loneliness and brain health [18]. Drawing from the UK Biobank, the most comprehensive biomedical dataset, we examined loneliness, brain volume, functional connectivity and structural connectivity in approximately 40,000 middle to older age adults. Using pattern-learning methods, we found that loneliness-linked neurobiological profiles converged once again on the default network. Patterns of regional grey matter volume in the default network were most robustly associated with loneliness. Positive loneliness associations were observed for functional connectivity within the default network. Further, lonely individuals had greater microstructural integrity of the fornix, a key fibre pathway that emerges from the hippocampal formation in the medial temporal lobe structures to send axons to directly connect to the ventromedial prefrontal cortex, a core default network region [25]. This striking convergence of loneliness associations with default network structure and function raises intriguing questions regarding the shifting impact of loneliness on the brain from young, through middle, and into older adulthood. While our interpretations remain preliminary, we hypothesize that prolonged experiences of loneliness into middle and late adulthood may result in the up-regulation of default network circuits that support mentalizing, reminiscence and imagination to fulfill desired but unmet needs for social interactions. Although speculative, this heightening of internally-directed cognitive processes may promote changes in vigilance and heightened emotional reactivity to external stimuli, also associated with the experience of loneliness [26]. We are now extending this work to explore longitudinal associations between loneliness and brain health in normative aging and individuals at elevated risk for AD. This will provide a broader lifespan perspective on the lifespan trajectory of ‘the lonely brain’.

While much work remains to be done, there is a growing urgency to understand links between age-related brain changes in the context of loneliness. According to a 2018 report from the American Association of Retired Persons, nearly one-third of individuals surveyed over age 45 reported feeling lonely. In just the last decade, the population of lonely people over age 45 has grown by more than 5 million, from 42.6 million to 47.8 million, and these data do not account for the global pandemic. Social distancing restrictions during the COVID-19 pandemic raise significant concerns about loneliness among older adults [27] and increases in loneliness have indeed been observed [28], with uncertain long-term consequences post-pandemic. Yet the experience of loneliness has clear implications for physical and mental health. Additionally, these impacts appear more acutely with advancing age. Surprisingly little data are currently available in humans that would illuminate how experiences of social isolation interact with brain structure and function in aging or brain disease. Reducing loneliness among older adults is a tractable public health issue. As the global pandemic has laid bare, public infrastructure to support connectedness is urgently needed. This is all the more the case in vulnerable populations, such as those in assisted living or Indigenous communities. Research progress is imperative to advance our understanding of the neural underpinnings of loneliness so that this knowledge may, in turn, inform public health policy, towards combating the oncoming pandemic of loneliness in older adulthood.
